# Sparse Representation-Based Extreme Learning Machine for Motor Imagery EEG Classification

**DOI:** 10.1155/2018/9593682

**Published:** 2018-10-28

**Authors:** Qingshan She, Kang Chen, Yuliang Ma, Thinh Nguyen, Yingchun Zhang

**Affiliations:** ^1^Institute of Intelligent Control and Robotics, Hangzhou Dianzi University, Hangzhou, Zhejiang 310018, China; ^2^Department of Biomedical Engineering, University of Houston, Houston, TX 77204, USA

## Abstract

Classification of motor imagery (MI) electroencephalogram (EEG) plays a vital role in brain-computer interface (BCI) systems. Recent research has shown that nonlinear classification algorithms perform better than their linear counterparts, but most of them cannot extract sufficient significant information which leads to a less efficient classification. In this paper, we propose a novel approach called FDDL-ELM, which combines the discriminative power of extreme learning machine (ELM) with the reconstruction capability of sparse representation. Firstly, the common spatial pattern (CSP) algorithm is adopted to perform spatial filtering on raw EEG data to enhance the task-related neural activity. Secondly, the Fisher discrimination criterion is employed to learn a structured dictionary and obtain sparse coding coefficients from the filtered data, and these discriminative coefficients are then used to acquire the reconstructed feature representations. Finally, a nonlinear classifier ELM is used to identify these features in different MI tasks. The proposed method is evaluated on 2-class Datasets IVa and IIIa of BCI Competition III and 4-class Dataset IIa of BCI Competition IV. Experimental results show that our method achieved superior performance than the other existing algorithms and yielded the accuracies of 80.68%, 87.54%, and 63.76% across all subjects in the above-mentioned three datasets, respectively.

## 1. Introduction

The brain-computer interface (BCI) is a system that allows its users to use their brain activity to control external devices which are independent of peripheral nerves and muscles [1, 2]. Motor imagery- (MI-) based sensorimotor rhythm (SMR) analysis, including mu (8–14 Hz) and/or beta (15–30 Hz) rhythms, recorded from the scalp over the sensorimotor cortex, is one of the widely used methods in the BCI field [3, 4]. However, these MI signals are highly nonstationary and inevitably contaminated with noise, and meanwhile, they strongly depend on subjects [5].

Sparse representation (SR), originally proposed by Olshausen et al. [6], attempts to simulate the working mechanism of primary visual cortex in the human visual system. The basic idea is to represent the data as a linear combination of atoms in a dictionary, whose requirement is that the coefficients are sparse, i.e., they contain only a small number of nonzero elements. In the last two decades, SR has been widely studied for reconstruction, representation, and compression of high-dimensional noisy data, such as computer vision, pattern recognition, and bioinformatics [7–9]. Recently, the SR techniques have also yielded promising results in the BCI systems [10–15]. Although SR is a powerful tool to reconstruct the originals from noisy and imperfect data, using the original training samples as the dictionary may not fully exploit the discriminative information hidden in the training samples. To address the problem, Yang et al. [16] proposed a Fisher discrimination dictionary learning (FDDL) framework to learn a structured dictionary and had good reconstruction capability for the training samples, yielding a 3.2% improvement over the sparse representation-based classification (SRC) algorithm on AR datasets in face recognition.

Recently, Huang et al. developed a new efficient learning algorithm called extreme learning machine (ELM) [17, 18] for training single-layer feedforward neural networks (SLFNs), featuring faster learning speed and better generalization capability in comparison with the well-known back propagation (BP) neural networks and support vector machines (SVMs). ELM has been applied to pattern recognition tasks in the BCI systems and has shown superior performance over traditional classification approaches [19–22]. In light of this advancement, efforts have been made in developing algorithms to integrate ELM and SR, thus exploiting the speed advantage and discriminative power of ELM and the antinoise performance and reconstruction ability of SR. A recent approach called extreme sparse learning (ESL) has been proposed in [23], which simultaneously learns sparse representation of the input signal and trains the ELM classifier. In the study by Yu et al. [24], the sparse coding technique is adopted to map the inputs to the hidden layer, instead of the random mapping used in classic ELM. Other ELM-SR hybrid models were also extensively studied, in which the ELM classifier is firstly employed to estimate noisy signals, and then a further identification for the estimated signals is carried out using the SRC algorithm [25–27].

Most of the existing ELM methods employ a single hidden layer network structure. While benefitting from a relatively fast training speed, it is well known that for a single hidden layer network, the training sample is always the original training sample set, which could limit the robustness of the network. Furthermore, due to its shallow architecture, feature learning using SLFNs may not be effective for natural signals (e.g., EEG). To incorporate a deeper network structure, a hierarchical-extreme learning machine (H-ELM) method has recently been developed, which allows for a layer-wise architecture design, and have shown to yield great classification performance [28]. In addition, multilayer structure has been extended into ELM in [29, 30] as well. Inspired by these works, we propose a new layer-wise structure framework called FDDL-ELM, which combines the idea of SR with ELM to learn a powerful nonlinear classifier. The proposed method first employs the Fisher discrimination criterion to learn a structured dictionary. With the learned dictionary, more discriminative sparse coding coefficients can be obtained, and more robust feature information can be extracted. Subsequently, the ELM classifier is utilized to discriminate the extracted features. The classification accuracy of the proposed method has been manifested by several benchmark datasets, as well as 2-class and 4-class real world EEG data from BCI Competition III Datasets IVa and IIIa and BCI Competition IV Dataset IIa.

The rest of the paper is organized as follows: Section 2 presents a brief introduction to basic ELM and FDDL and provides detailed description of the proposed FDDL-ELM algorithm.  Section 3 evaluates the performance of the FDDL-ELM method through a series of experiments on several benchmark datasets, as well as motor imagery EEG datasets. Finally, we will conclude the paper and present some future work in Section 4.

## 2. Methodology

### 2.1. Classic ELM

ELM was originally implemented for single-hidden layer feedforward neural networks and then extended to generalize feedforward networks. By using random hidden node parameters and tuning-free strategy, ELM has some notable advantages, such as easy implementation, fast learning speed, and superior generalization performance [17], thus making it a suitable choice for the recognition problem of EEG signals in different motor imagery tasks.

Consider a dataset containing *N* training samples, {**X**, **Y**}={**x**_*i*_, **y**_*i*_}, *i*=1,2, ⋯, *N*, with the input **x**_*i*_=[*x*_*i*1_, *x*_*i*2_,…,*x*_*ip*_]^T^ ∈ *R*^*p*^ and its corresponding desired output **y**_*i*_=[*y*_*i*1_, *y*_*i*2_,…,*y*_*iq*_]^T^ ∈ *R*^*q*^, where T denotes a transpose operation. Assuming that *m* is the number of hidden neurons, and *g*(·) is the activation function, the output function of ELM is mathematically modeled as

(1) $$\begin{array}{c} y_{j}=\overset{m}{\underset{i=1}{\sum }}\beta_{i}g\left(a_{i}^{\text{T}}x_{j}+b_{i}\right),\;j=1,2,\cdots ,N, \end{array}$$

where **β**_*i*_=[*β*_*i*1_, *β*_*i*2_, ⋯,*β*_*iq*_]^T^ is the weight vector that connects the *i* − th hidden neuron and the output neurons, **a**_*i*_=[*a*_*i*1_, *a*_*i*2_, ⋯,*a*_*i*p_]^T^ is the randomly chosen input weight vector connecting the *i* − th hidden neuron and the input neurons, *b*_*i*_ is the randomly chosen bias of the *i* − th hidden node, and *y*_*j*_ is the actual output corresponding to input *x*_*j*_.

For convenience of expression, the Equation (1) is written in matrix notation as

(2) $$\begin{array}{c} Y=H\beta , \end{array}$$

where **Y**=[*y*_1_, *y*_2_, ⋯,*y*_*N*_]_*N*×*q*_^T^ is the expected network output, **β**=[**β**_1_, **β**_2_, ⋯,**β**_*m*_]_*m*×*q*_^T^ denotes the weight of output layer, and **H** is the hidden layer output matrix which is defined as

(3) $$\begin{array}{c} H={\left[\begin{array}{ccc} g\left(a_{1}^{\text{T}}x_{1}+b_{1}\right) & \ldots & g\left(a_{m}^{\text{T}}x_{1}+b_{m}\right)\\ \ldots & \ldots & \ldots \\ g\left(a_{1}^{\text{T}}x_{N}+b_{1}\right) & \ldots & g\left(a_{m}^{\text{T}}x_{N}+b_{m}\right) \end{array}\right]}_{N\times m}. \end{array}$$

To have better generalization performance, the regularization parameter *C* is introduced in [19], and its corresponding objective function is given by

(4) $$\begin{array}{c} \underset{\beta }{\text{arg min}}\left({\left\|\beta \right\|}_{2}^{2}+C{\left\|H\beta -Y\right\|}_{2}^{2}\right), \end{array}$$

where ‖·‖_2_ denotes the *l*_2_-norm of a matrix or a vector. We can obtain the output weight vector **β** using the Moore–Penrose principle. The solution of Equation (4) is **β**=(**I**/*C*+**H**^T^**H**)^−1^**H**^T^**Y** if *N* > *m* and **β**=**H**^T^(**I**/*C*+**HH**^T^)^−1^**Y** if *N* < *m*.

### 2.2. Fisher Discrimination Dictionary Learning

Sparse representation-based classification (SRC) was proposed for face recognition, which directly used the training samples of all classes as the dictionary to code the query face image and classified it by evaluating which class leads to the minimal reconstruction error [7]. However, the dictionary in use may not be effective enough to represent the query images due to the uncertain and noisy information in the original training images, and the discriminative information hidden in the training samples is not sufficiently exploited by such a naïve-supervised dictionary learning approach [16]. To address these problems, the FDDL method is proposed, utilizing both the discriminative information in the reconstruction error and sparse coding coefficients.

Denote **A**=[**A**_1_, **A**_2_, ⋯, **A**_*c*_] as the training set, where **A**_*i*_ is the subset of the training samples from class *i*, and *c* is the total number of classes, and an overcomplete dictionary **D**=[**D**_1_, **D**_2_, ⋯, **D**_*c*_], where **D**_*i*_ is the class-specified subdictionary associated with class *i*. Let **X** be the coding coefficient matrix of **A** over **D**, we can write **X** as **X**=[**X**_1_, **X**_2_, ⋯, **X**_*c*_], where **X**_*i*_ is the submatrix containing the coding coefficients of **A**_*i*_ over **D**. The objective function is written as follows:

(5) $$\begin{array}{c} J_{\left(D,X\right)}=\arg\underset{D,X}{\min}\left\{r\left(A,D,X\right)+\lambda_{1}{\left\|X\right\|}_{1}+\lambda_{2}f\left(X\right)\right\}, \end{array}$$

where *r*(**A**, **D**, **X**) is the discriminative fidelity term, ‖**X**‖_1_is the sparse constraint in which the notation ‖·‖_1_ denotes the *l*_1_-norm, *f*(**X**) is a discrimination constraint, and *λ*_1_ and *λ*_2_ are the scalar parameters.

(6) $$\begin{array}{c} r\left(A_{i},D,X_{i}\right)={\left\|A_{i}-DX_{i}\right\|}_{F}^{2}+{\left\|A_{i}-D_{i}X_{i}^{i}\right\|}_{F}^{2}+\overset{c}{\underset{j=1,j\neq i}{\sum }}{\left\|D_{j}X_{i}^{j}\right\|}_{F}^{2}, \end{array}$$

where ‖·‖_*F*_ means the *F*-norm, **X**_*i*_^*i*^ is the coding coefficient of **A**_*i*_ over the subdictionary **D**_*i*_, and **X**_*i*_^*j*^ is the coding coefficient of **A**_*i*_ over the subdictionary **D**_*j*_. The minimization of *r*(**A**, **D**, **X**) means that the reconstruction error of the *i* − th class of samples is minimized, and the reconstruction error through the *i* − th subdictionary is also minimized while the reconstruction by other subclass dictionaries should be minimized. Its purpose is to ensure that the reconstruction error constraint is minimized, and the sparse coefficient can be more discriminative.

*f*(**X**) is a discriminative coefficient term which is given in the following:

(7) $$\begin{array}{c} f\left(X\right)=\text{tr}\left(S_{\text{W}}\left(X\right)\right)-\text{tr}\left(S_{\text{B}}\left(X\right)\right)+\eta {\left\|X\right\|}_{F}^{2}, \end{array}$$

where tr(·) indicates the trace of subspace, *S*_W_(**X**) is the within-class scatter of **X**, *S*_B_(**X**) is the between-class scatter of **X**, and *η* is a parameter.

(8) $$\begin{array}{c} S_{\text{W}}\left(X\right)=\overset{c}{\underset{i=1}{\sum }}\underset{x_{k}\in X_{i}}{\sum }\left(x_{k}-m_{i}\right){\left(x_{k}-m_{i}\right)}^{\text{T}},\\ S_{\text{B}}\left(X\right)=\overset{c}{\underset{i=1}{\sum }}n_{i}\left(m_{i}-m\right){\left(m_{i}-m\right)}^{\text{T}}, \end{array}$$

where **m**_*i*_ and **m** are the mean vectors of **X**_*i*_ and **X** respectively, and *n*_*i*_ is the number of samples in class **A**_*i*_.

### 2.3. The Proposed FDDL-ELM Method

**Figure 1:**
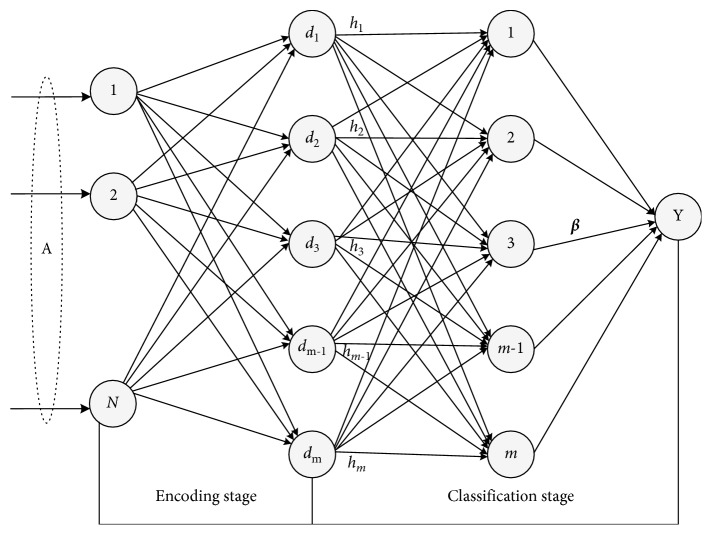
A schematic for the overall framework of the FDDL-ELM-learning algorithm.

In this section, we propose a novel nonlinear classification model that rests on a new ELM framework for multilayer perceptron (MLP), named FDDL-ELM. The framework consists of two stages: an encoding stage and a classification stage. The former stage uses the FDDL approach to map the input features into a midlevel feature space, and then the ELM algorithm is performed for final decision making in the latter stage. The framework of FDDL-ELM is shown in Figure 1.

Let **A** be an input containing *N* training samples {**A**, **Y**} with **A**=[**A**_1_, **A**_2_, ⋯, **A**_*c*_], and **Y** is the corresponding desired output, where **A**_*i*_ is the subset of the input from class *i*, and *c* is the total number of classes.

Step (1): utilize the FDDL algorithm to learn a structured dictionary **D**.

By incorporating Equations (6) and (7) into Equation (5), the objective function is rewritten as

(9) $$\begin{array}{c} J_{\left(D,X\right)}=\arg\underset{\left(D,X\right)}{\min}\left\{\overset{c}{\underset{i=1}{\sum }}r\left(A_{i},D,X_{i}\right)+\lambda_{1}{\left\|X\right\|}_{1}+\lambda_{2}\left(\text{tr}\left(S_{\text{W}}\left(X\right)-S_{\text{B}}\left(X\right)\right)+\eta {\left\|X\right\|}_{F}^{2}\right)\right\}. \end{array}$$

The optimization of the objective function consists of two steps: First, update **X** by fixing **D**, and then update **D** while fixing **X**. The procedures are iteratively implemented for the desired discriminative dictionary **D** and the discriminative coefficients **X** as done in [16].

Step (2): reconstruct the signals for the high-level sparse feature information.

With the desired dictionary **D** and the coefficients **X** in the Step (1), we can get the reconstructed signals **B** which can uncover important information hidden in the original signals and is simplified as follows:

(10) $$\begin{array}{c} B=DX. \end{array}$$

Step (3): discriminate the reconstructed signals **B** using the ELM classification method.

1. Randomly generate the hidden node parameters (**a**_*i*_, *b*_*i*_) for *i*=1,2, ⋯, *m*.
2. The new hidden-layer output matrix **G** can be written as

(11) $$\begin{array}{c} G={\left[\begin{array}{ccc} g\left(a_{1}^{\text{T}}DX_{1}+b_{1}\right) & \ldots & g\left(a_{m}^{\text{T}}DX_{1}+b_{m}\right)\\ \ldots & \ldots & \ldots \\ g\left(a_{1}^{\text{T}}DX_{N}+b_{1}\right) & \ldots & g\left(a_{m}^{\text{T}}DX_{N}+b_{m}\right) \end{array}\right]}_{N\times m}. \end{array}$$

(3) The regularization parameter *C* is introduced, and the output weight ***β*** is calculated as follows:

(12) $$\begin{array}{c} \beta ={\left(\frac{I}{C}+G^{\text{T}}G\right)}^{-1}G^{\text{T}}Y,\;\text{for}N>m,\\ \beta =G^{\text{T}}{\left(\frac{I}{C}+GG^{\text{T}}\right)}^{-1}Y,\;\text{for}N<m. \end{array}$$

Extensive efforts have been paid to the optimal selection of *C* and the leave-one-out (LOO) cross-validation strategy combining with the predicted residual sum of squares (PRESS) statistic is one of the most effective methods [26].

Step (4): For test data {**A**_test_, **Y**_test_} and the learned dictionary **D**, we can reconstruct the **A**_test_ in the encoding stage and then calculate the labels **Y**_predict_ using the ELM classifier.

## 3. Experimental Results and Discussion

In this section, several experiments on benchmark datasets and EEG datasets were performed to evaluate the performance of the proposed FDDL-ELM method, as compared with the other state-of-the-art approaches. All methods were implemented using MATLAB 2014b environment on a computer with a 2.6 GHz processor and 8.0 GB RAM.

### 3.1. Experiment on Benchmark Datasets

#### 3.1.1. Description

In order to evaluate its performance, the proposed FDDL-ELM method was first applied to four popular benchmark datasets in the UCI repository [31]. The details of these datasets are shown in Table 1.

The Liver Disorders dataset is a medical application, which consists of 345 samples belonging to 2 categories, and each sample extracts 6 features for representation. The Diabetes dataset contains 768 samples belonging to two categories. For each sample, 8 features are extracted. The Waveform dataset consists of 5000 samples from 3 classes of noisy waveforms, and each sample contains 21 attributes. The Columbia Object Image Library (COIL-20) is a multiclass image classification dataset and consists of 1440 grayscale image sample of 20 different objects, in which each sample is a 32 × 32 grayscale image of one object taken from a specific view.

#### 3.1.2. Experimental Setup

In Table 1, the column “Random perm” denotes whether the training and test data are randomly assigned. In each data partition, the ratio between training and test sample is 1 : 1. The classification process was repeated ten times, and the average of these outcomes was the final classification rate.

This proposed FDDL-ELM algorithm has 5 tuning parameters: *λ*_1_, *λ*_2_, and *η* in the encoding stage, as well as the number of hidden nodes *m*, and the regularization parameter *C* in the classification stage. In all the experiments, the optimal parameters *λ*_1_ and *λ*_2_ are searched using five-fold cross-validation from a small set {0.001, 0.005, 0.01, 0.05, 0.1}, and *η* is set to 1, as done in [16]. The optimal parameters *m* and *C* were determined from *m* ∈ {100,200, ⋯, 1500} and *C* ∈ {e^−5^, e^−4^, ⋯, e^5^} using the LOO cross-validation strategy based on the minimum MSE^PRES^ [27]. It is noted that *C* is automatically chosen and not fixed during the process of repeating ten times in the classification stage. The settings of these tuning parameters for four benchmark datasets are summarized in Table 2.

#### 3.1.3. Comparisons with Other State-of-the-Art Algorithms

In this experiment, we compare the proposed FDDL-ELM with three baseline algorithms, including ELM, FDDL, and H-ELM. The classification performance is evaluated in terms of average accuracy and standard deviation (acc ± sd).  Table 3 summarizes the performance results using four methods on the benchmark datasets.

From the results shown in Table 3, it is evidenced that the FDDL-ELM algorithm achieved comparable performance with other state-of-the-art methods, such as single-layer ELM, FDDL, and H-ELM with deep architecture. For the Diabetes dataset, the FDDL-ELM approach achieved more than 9% improvement over FDDL. For the Liver Disorders dataset, although the average classification accuracy of H-ELM (74.01%) was better than that of FDDL-ELM (72.38%), the accuracy of FDDL-ELM was higher than ELM by 0.23% and FDDL by 6.61%. For the Waveform dataset, the FDDL-ELM approach yielded a mean accuracy of 85.02%, a 0.57% improvement over ELM, and a 0.30% improvement over H-ELM. The average classification accuracy of COIL-20 dataset obtained by FDDL-ELM was 98.33%, higher than those of ELM (96.14%) and H-ELM (97.13%). Based on these observations, the proposed FDDL-ELM approach outperformed the original ELM and FDDL methods on all four datasets and had comparable performance on most of the four datasets compared with H-ELM.

#### 3.1.4. The Impact of the Parameters

There are five parameters in our algorithm: *λ*_1_, *λ*_2_, *η*, *m*, and *C*. Since *η* is set to 1 [16], and *C* is automatically chosen [27], we will investigate the impact of the other three parameters (*λ*_1_, *λ*_2_, and *m*) on the performance of our algorithm in this section. *λ*_1_ and *λ*_2_ are respectively changed among {0.001, 0.005, 0.01, 0.05, 0.1}. The parameter *m* decides the number of hidden neurons, and its value is selected among {100,200, ⋯, 1500}.

**Figure 2:**
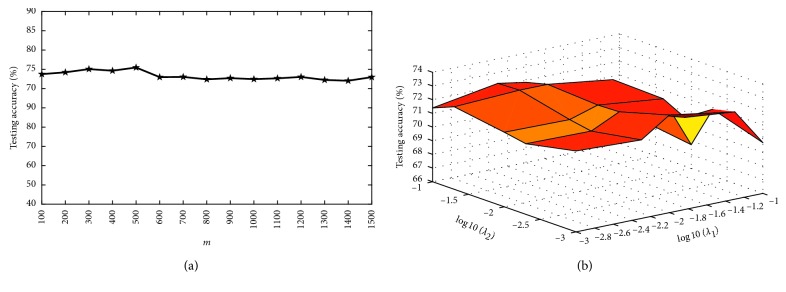
Testing accuracy with different parameters on Diabetes. (a) Accuracy in terms of *m*; (b) accuracy curve in terms of (*λ*_1_, *λ*_2_).

**Figure 3:**
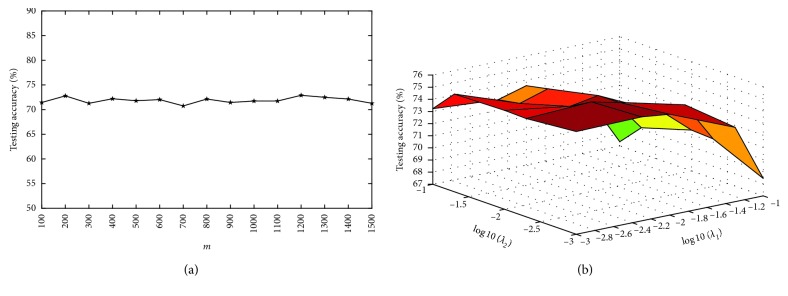
Testing accuracy with different parameters on liver disorders. (a) Accuracy in terms of *m*; (b) accuracy curve in terms of (*λ*_1_, *λ*_2_).

**Figure 4:**
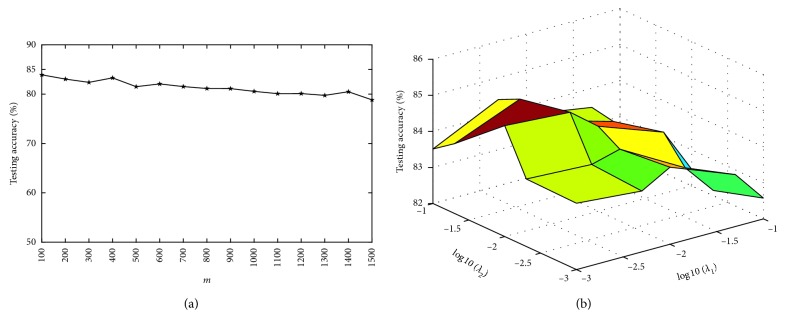
Testing accuracy with different parameters on waveform. (a) Accuracy in terms of *m*; (b) accuracy curve in terms of (*λ*_1_, *λ*_2_).

**Figure 5:**
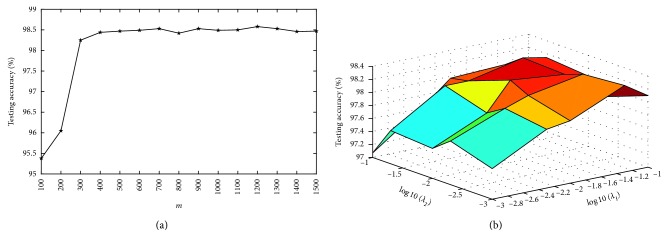
Testing accuracy with different parameters on COIL-20. (a) Accuracy in terms of *m*; (b) accuracy curve in terms of (*λ*_1_, *λ*_2_).

Figures 2(a)–5(a) show the testing results of our algorithm as the parameter *m* changes on four datasets: Diabetes, Liver Disorders, Waveform, COIL-20 datasets, respectively. As can be seen, the performance of our algorithm is relatively stable with respect to *m*. Figures 2(b)–5(b) give the plots of testing accuracies as *λ*_1_ and *λ*_2_ vary on four datasets, respectively. From these results, it can be observed that the performance is more pronouncedly affected by the parameters *λ*_1_ and *λ*_2_, relative to *m*. For a tradeoff between the classification performance and computation complexity, we can use a comparatively small number of nodes *m* when selecting *λ*_1_ and *λ*_2_ in real EEG applications.

### 3.2. Experiment on BCI Datasets

#### 3.2.1. Description

This section evaluates the performance of the proposed FDDL-ELM method on MI EEG datasets. There are three datasets for analysis, including two datasets for binary classification and one dataset for multiclassification, as described below:

1. Dataset IVa, BCI competition III [32]: this dataset contains EEG signals from 5 subjects, who performed 2-class MI tasks: right hand and foot. EEG signals were recorded using 118 electrodes. A training set and a testing set were available for each subject. Their size was different for each subject. More precisely, 280 trials were available for each subject, among which 168, 224, 84, 56, and 28 composed the training set for subjects A1, A2, A3, A4, and A5, respectively, and the remaining trials composed the testing set.
2. Dataset IIIa, BCI competition III [33]: this dataset comprised EEG signals from 3 subjects who performed left hand, right hand, foot, and tongue MI. EEG signals were recorded using 60 electrodes. For the purpose of binary classification, only 2-class EEG signals (left and right hand MI) were used as done in [34]. Both the training and testing sets were available for each subject. Both sets contain 45 trials per class for subject B1, and 30 trials per class for subjects B2 and B3.
3. Dataset IIa, BCI competition IV [35]: this dataset consists of EEG signals from 9 subjects who performed 4-class MI tasks: left hand, right hand, foot, and tongue MI. EEG signals were recorded using 22 electrodes. The training and testing sets contain 288 trials for each class, respectively.

#### 3.2.2. Experimental Setup

Data preprocessing was first performed on the raw EEG data. In particular, for each trial, we extracted features from the time-segment spanning from 0.5 s to 2.5 s after the cue instructing the subject to perform MI. Each trial was band-pass filtered in 8–30 Hz, using a fifth-order Butterworth filter. Next, the dimension of the EEG signal was reduced using the common spatial pattern (CSP) algorithm, a widely used feature selection method for MI-based BCIs [12, 34]. Finally, the filtered EEG signals by CSP were discriminated by different classification methods in our experiment.

In this work, the classification process was repeated ten times, and the average accuracy was recorded for further analysis. The selection process of the parameters *λ*_1_, *λ*_2_, *η*, and *C* were the same as those described in Section 3.1.2, and the setting of the hidden node *m* was {10,20, ⋯, 100}.

#### 3.2.3. Comparisons with Related Algorithms

We compared the proposed method FDDL-ELM with ELM, FDDL, and H-ELM on BCI Competition III Datasets IVa and IIIa and BCI Competition IV Dataset IIa. The average classification accuracies of all four algorithms are shown in Table 4.

From Table 4, it can be seen that the proposed method outperformed the ELM and FDDL algorithms on almost all subjects (except subject B2) in binary-classification applications. For subject B2, the ELM method obtained the average accuracy of 68.33%, a 0.33% improvement over FDDL-ELM. Compared with H-ELM which adopts a deep architecture, FDDL-ELM yielded comparable performance on all the 8 subjects and especially performed better in 4 subjects (A2, A3, A4, and B2). Furthermore, the proposed algorithm yielded the highest average accuracy on Datasets IVa and IIIa. For the Dataset IVa, the FDDL-ELM approach achieved a mean accuracy of 80.68%, a 0.73% improvement over ELM, and a 1.35% improvement over H-ELM. Moreover, a paired *t*-test revealed no significant difference between the FDDL-ELM and H-ELM approaches (*p*  =  0.626) and a significant difference between the FDDL-ELM and ELM approaches (*p*  =  0.04). For the Dataset IIIa, the average classification accuracy obtained by FDDL-ELM was 87.54%, higher than that of ELM (87.35%), FDDL (83.26%), and H-ELM (85.63%). Furthermore, a paired *t*-test revealed no significant difference between the FDDL-ELM and H-ELM approaches (*p*  =  0.596). These results have shown that the FDDL-ELM method has achieved a great classification capacity in binary-classification applications.

In the 4-class-classification application for BCI Competition IV Dataset IIa, the average classification accuracies for the 9 subjects using four algorithms are also shown in Table 4. Note that our method also outperformed ELM and FDDL in 8 of the 9 subjects (except subject C8). For subject C8, ELM gained the best result (81.87%) compared with FDDL (63.72%), FDDL-ELM (80.60%), and H-ELM (76.46%). The FDDL-ELM approach performed the best in 4 subjects (C1, C3, C6, and C7), whereas H-ELM achieved the best result in 4 subjects (C2, C4, C5, and C9). The average classification accuracy of 9 subjects using FDDL-ELM was 63.76%, slightly lower than H-ELM (64.46%). A paired *t*-test revealed no significant difference between the FDDL-ELM and H-ELM approaches (*p*  =  0.519) and a significant difference between the FDDL-ELM and FDDL approaches (*p* is less than 0.01). These results showed that when compared to the H-ELM algorithm, our method can achieve similar results without the deep architecture.

In these experiments, the proposed FDDL-ELM method exhibited an excellent performance in both binary-classification and multiclassification cases. The nonlinear property of FDDL-ELM allowed for its superior performance over the FDDL approach when processing the nonstationary EEG signals. Furthermore, FDDL-ELM is more suitable in analyzing noisy EEG data than basic ELM because its encoding stage can acquire a higher representation of the raw signals and extract more effective feature information. Compared with the H-ELM, an algorithm with a deep architecture design, our method also yielded comparable results. In particular, on the binary-classification datasets (BCI Competition III Datasets IVa and IIIa), our method gained higher average accuracies (80.68% and 87.54%) than that of H-ELM (79.33% and 85.63%), respectively.

## 4. Conclusion

In this paper, we have proposed a new ELM framework called FDDL-ELM, which achieves a sparse representation of input raw data with layer-wise encoding, while still benefiting from the universal approximation capability of the original ELM. We verified the generalizability and capability of FDDL-ELM using publicly available benchmark databases and MI-BCI datasets. In these applications, FDDL-ELM demonstrated superior classification performance than the other relevant state-of-the-art methods. However, there are still several questions to be further investigated in future work. The nonstationary nature of EEG signals means that a classification model built earlier using the previous data is not able to well reflect the changes that have already taken place to the signals. Consequently, the online updates to the classification model are needed. Recently, the ensemble of subset online sequential extreme learning machine (ESOS-ELM) method is proposed for class imbalance learning [36]. In addition, an online sequential extreme learning machine with kernels (OS-ELMK) has been proposed for prediction of nonstationary time series [37]. In the study by Mirza et al.[38], a multilayer online sequential extreme learning machine has been proposed for image classification. In the future work, we will investigate the online learning algorithm of FDDL-ELM for analyzing MI EEG signals.

## Figures and Tables

**Table 1:** Description of the benchmark datasets.

Datasets	Training	Testing	Features	Classes	Random perm
Liver Disorders	172	172	6	2	Yes
Diabetes	384	384	8	2	Yes
Waveform	2500	2500	21	3	Yes
COIL-20	720	720	1024	20	Yes

**Table 2:** Parameter settings of FDDL-ELM on the benchmark datasets.

Datasets	*λ*_1_	*λ*_2_	*m*
Liver Disorders	0.01	0.001	400
Diabetes	0.005	0.05	500
Waveform	0.001	0.01	100
COIL-20	0.05	0.001	900

**Table 3:** Comparisons of classification results on each dataset using different methods.

Method	Liver Disorders	Diabetes	Waveform	COIL-20
	Acc ± sd	Acc ± sd	Acc ± sd	Acc ± sd
ELM	72.15 ± 1.54	74.22 ± 1.10	84.45 ± 0.69	96.14 ± 1.03
FDDL	65.77 ± 4.45	65.43 ± 2.49	79.18 ± 1.19	96.48 ± 0.85
H-ELM	**74.01** **±** **0.87**	71.67 ± 1.26	84.72 ± 0.30	97.13 ± 0.71
FDDL-ELM	72.38 ± 1.49	**75.33** **±** **0.68**	**85.02** **±** **0.32**	**98.33** **±** **0.62**

**Table 4:** Comparisons of classification results on 2-class and 4-class BCI datasets using different methods.

Datasets		Methods			
		ELM	FDDL	FDDL-ELM	H-ELM
Dataset IVa	A1	60.71	57.50	61.70	**63.39**
	A2	100	84.29	**100**	98.39
	A3	73.37	70.51	**73.88**	64.08
	A4	86.61	65.00	**88.17**	85.67
	A5	79.05	77.14	79.64	**85.16**
	Mean	79.95	70.89	**80.68**	79.33

Dataset IIIa	B1	96.89	93.11	97.78	**98.56**
	B2	**68.33**	60.33	68.00	60.00
	B3	96.83	96.33	96.83	**98.33**
	Mean	87.35	83.26	**87.54**	85.63

Dataset IIa	C1	76.43	62.77	**76.74**	75.69
	C2	45.38	32.92	45.70	**47.74**
	C3	76.32	70.76	**77.13**	76.98
	C4	59.24	45.59	60.50	**61.84**
	C5	37.12	31.42	36.24	**37.85**
	C6	45.90	35.28	**47.57**	47.08
	C7	78.99	66.91	**80.30**	80.07
	C8	**81.87**	63.72	80.60	76.46
	C9	67.36	65.48	69.10	**76.42**
	Mean	63.18	52.76	63.76	**64.46**

## Data Availability

Three datasets were employed in this study, including two datasets for binary classification and one dataset for multiclassification, which are publicly available: (1) dataset IVa, BCI competition III [31]: this dataset contains EEG signals from 5 subjects, who performed 2-class MI tasks: right hand and foot. (2) Dataset IIIa, BCI competition III [34]: this dataset comprised EEG signals from 3 subjects who performed left hand, right hand, foot, and tongue MI. (3) Dataset IIa, BCI competition IV [32]: this dataset consists of EEG signals from 9 subjects who performed 4-class MI tasks: left hand, right hand, foot, and tongue MI.
